# Indoleacetic Acid Levels in Wheat and Rice Seedlings under Oxygen Deficiency and Subsequent Reoxygenation

**DOI:** 10.3390/biom10020276

**Published:** 2020-02-11

**Authors:** Vladislav V. Yemelyanov, Victor V. Lastochkin, Tamara V. Chirkova, Sylvia M. Lindberg, Maria F. Shishova

**Affiliations:** 1 Department of Genetics and Biotechnology, Saint-Petersburg State University, Universitetskaya em., 7/9, 199034 Saint-Petersburg, Russia; 2 Department of Plant Physiology and Biochemistry, Saint-Petersburg State University, Universitetskaya em., 7/9, 199034 Saint-Petersburg, Russia; 3 Department of Ecology, Environment and Plant Sciences, Stockholm University, SE-106 91, Stockholm, Sweden

**Keywords:** auxin, oxygen deficiency, post-anoxia, rice, tobacco, wheat

## Abstract

The lack of oxygen and post-anoxic reactions cause significant alterations of plant growth and metabolism. Plant hormones are active participants in these alterations. This study focuses on auxin–a phytohormone with a wide spectrum of effects on plant growth and stress tolerance. The indoleacetic acid (IAA) content in plants was measured by ELISA. The obtained data revealed anoxia-induced accumulation of IAA in wheat and rice seedlings related to their tolerance of oxygen deprivation. The highest IAA accumulation was detected in rice roots. Subsequent reoxygenation was accompanied with a fast auxin reduction to the control level. A major difference was reported for shoots: wheat seedlings contained less than one-third of normoxic level of auxin during post-anoxia, while IAA level in rice seedlings rapidly recovered to normoxic level. It is likely that the mechanisms of auxin dynamics resulted from oxygen-induced shift in auxin degradation and transport. Exogenous IAA treatment enhanced plant survival under anoxia by decreased electrolyte leakage, production of hydrogen peroxide and lipid peroxidation. The positive effect of external IAA application coincided with improvement of tolerance to oxygen deprivation in the 35S:*iaaM* × 35S:*iaaH* lines of transgene tobacco due to its IAA overproduction.

## 1. Introduction

Plant growth is a complicated process under the control of multiple internal and external effectors. Plant response is always the sum of positive and negative alterations appearing at molecular, cell, tissue and organism levels. Nowadays modeling provides an efficient way to elucidate systemic plant response [1]. Each of the stressors has peculiar mechanisms of action involving activation of specific gene groups that trigger specific developmental, physiological and metabolic adaptations. Nevertheless, accumulated data suggest that there are possible integrative responses common for a group of factors, such as drought, salinity and chilling stress [2]. Here we aim to reveal some cross-adaptations to oxygen deficiency and further reoxygenation.

Higher plants strongly depend on ambient oxygen concentration. The plant facilities to survive oxygen deprivation have been studied for decades [3,4,5,6,7]. Partial (hypoxia) or complete lack of oxygen (anoxia) characterizes both natural and agricultural ecosystems and has a serious economic impact [8,9]. Global climate changes make increasingly relevant studies focused on mechanisms that ensure survival, growth and development of plants and minimize yield loss.

Resistance of plants to oxygen deficiency depends on a set of developmental, physiological and metabolic adaptations. There are two contrasting strategies behind such adaptations, low oxygen escape syndrome (LOES) and low oxygen quiescence syndrome (LOQS) [3]. The first one is based on a rapid shoot growth, hyponastic bending of leaves, formation of aerenchyma, initiation of adventitious roots, and alteration of leaf anatomy to improve the diffusion of gases, etc. [4,5,6]. This is a strategy to avoid oxygen deprivation by facilitating gas exchange between the submerged and aerated organs of plant. The quiescent strategy consists of fast growth termination and redirection of stored energy and carbohydrates in order to survive in an oxygen-deficient environment and enables growth recovery when normoxic conditions are restored [4,5,6]. ATP generated due to starch mobilization, glycolysis, and fermentation is used predominantly for the synthesis of proteins involved in energy metabolism, membrane transport, elimination of reactive oxygen species (ROS), and chaperone activity [5,6,7,9].

Both strategies are intensively regulated by phytohormones [10]. Ethylene plays a crucial role in plant response to oxygen deprivation [2,11,12]. Submergence induces synthesis and physical entrapment of ethylene inside plant tissues [5,13]. The ethylene accumulation activates a group of transcription factors—ethylene responsive factor VII (ERF-VII). The representatives of this group SNORKEL1 (SK1) and SNORKEL2 (SK2) in rice control escape strategy (LOES). They alter hormonal balance by accumulating gibberellins and decreasing abscisic acid leading to a fast internode elongation [14]. SKs are responsible for regulating carbohydrate breakdown for the provision of accelerated growth via activation of a number of protein kinases and transcription factors [12]. The quiescent strategy (LOQS) in rice is governed by SUBMERGENCE 1A (SUB1A, also belonging to ERF-VII). SUB1A suppresses ethylene and GA signaling [5], positively regulating the transcription of genes related to the fermentation and repressing of genes involved in carbohydrates metabolism [15]. 

In *Arabidopsis thaliana*, the ERF-VII factors also participate in the regulation of the anoxic response. Three factors RAP2 (RELATED TO AP2) - RAP2.2 (ERF75), RAP2.3 (ERF72/EBP) and RAP2.12 (ERF74) are constitutively synthesized during normoxia and elevated by ethylene [9]. Two other factors HRE (HYPOXIA RESPONSIVE ERF) - HRE1 (ERF73) and HRE2 (ERF71) are actively induced under oxygen deficiency [5]. These ERF-VII factors in the presence of oxygen very quickly pass proteolysis according to the N-terminus rule, while at low oxygen these TFs are stabilized and induce hypoxic gene expression [5,12,16].

It is interesting that even at normoxia there are few examples of “hypoxic niches” existing inside the native plant body. One example is the uncontrolled cell proliferation of crown galls. Development of internal hypoxic conditions triggers the low-oxygen response pathway, mediated by ERF-VII group [17]. Another example is lateral root primordia. It has been shown that the ethylene-induced transcription factors of the ERF-VII group control root architecture. They decrease the expression of auxin-induced genes *LBD16*, *LBD18* and *PUCHI*, encoding transcription factors essential for lateral root development [18].

Ethylene–auxin crosstalk is the major player that initiates stem hypertrophy and formation of adventitious roots during submergence in different plants [5,10,13] via ethylene-dependent redistribution of auxin transport [19,20], accumulation [20,21,22,23] and increase in sensitivity to auxin (indoleacetic acid, IAA) in root-forming tissues [24]. Flooding induced hyponastic response of *Rumex palustris* petioles depending on auxin [25]. Epinasty in susceptible plants like tomato also depends on IAA–ethylene interactions [26]. Auxin affects growth during oxygen deficiency in a wide spectrum of wetland plants. It stimulates growth in leaves of the fern *Regnellidium diphyllum* [27], stems of *Potamogeton pectinatus* [28], petioles of *Nymphoides peltata* [29], *Ranunculus sceleratus* [30,31,32], and *R. palustris* [33]. On the other hand, auxin is not required for the ethylene-mediated submergence-induced growth of coleoptiles [34] and internodes of the most studied LOES plant, the deep-water rice [35]. Under complete anoxia, IAA has no effect on the growth of excised coleoptiles [36]. 

Alterations of endogenous IAA level also vary in different plants during oxygen shortage. Submergence reduces total level of auxin in rice coleoptiles [37] and in petioles of *R. palustris* [25]. Wheat that is more susceptible to hypoxia shows less IAA increase under oxygen lack than the more tolerant oat [38,39]. Rice seedlings accumulated even higher amounts of IAA at total anoxia [40,41]. However, it is still unclear, what is the mechanism of auxin increase under oxygen deprivation, and how it affects plant organ/tissue tolerance. 

An additional factor should be taken into consideration. The reestablishment of normal oxygen levels in the natural environment after hypoxia or anoxia provokes intensive oxidative damage to plants affecting growth and survival. Fast reentry of oxygen leads to an oxidation of reduced intermediate and end-products that accumulate during preceding anoxia, soil toxins, overproduction of ROS and other toxic oxidative products [6,12,42]. 

ROS accumulation is well documented for wide spectra of abiotic interactions [43]. The origin of ROS in plant cell is different. The cell wall, plasma membrane, cytosol, mitochondria, chloroplasts, peroxisomes, glyoxysomes, and endoplasmic reticulum are known to participate in ROS production [44]. An important ROS source in leaf tissues is photosynthesis. Alterations in the photochemical efficiency of PSII in rice leaves were found specific for different stress factors such as high light, salinity, high osmolality, and heavy metal stress [45]. Alternatively, ROS generation is related to respiration and oxidative processes in plasma membrane/apoplast and peroxisomes [43,44]. These two mechanisms are supposed to be responsible for ROS elevation in roots. Surprisingly ROS accumulation was mentioned even under oxygen deprivation [46]. Some phospholipases D are also involved in ROS signaling under hypoxic stress [47]. 

In plant cells ROS degradation can be implemented by low molecular weight antioxidants (ascorbate, glutathione, tocopherol) and detoxifying enzymes (superoxide dismutases, catalases, and peroxidases). Many genes responsible for ROS utilization and antioxidant defense are triggered by hypoxia [48]. During reaeration, the immediate repletion of oxygen enhances the Arg/N-terminal pathway of ERF-VIIs degradation via plant Cys-oxidases, leading to the fast repression of anaerobic mRNAs’ transcription. On the other hand, in *A. thaliana*, reoxygenation promotes accumulation of transcripts associated with ethylene production [5]. This suggests that ethylene signaling plays a role during oxygen depletion stress recovery. Auxin is an important plant hormone regulating growth and development. Therefore, it may be involved in plant survival and recovery in the post-anoxic period.

Taken together the provided data reveal a complicated link between hypoxia trigged ethylene and auxin crosstalk. Different effects of auxin on shoot elongation and root architecture under oxygen deficiency might result from different levels of plant-organ sensitivity to oxygen deprivation and to multiple elements of auxin signaling and the systems related to its metabolism. It is questionable as to whether auxin itself has a direct effect on plant growth and biochemical adaptation to oxygen deficiency. Even more puzzling is the role auxin plays under increased levels of ROS concentration in plant tissues during post-hypoxic/-anoxic reoxygenation. This study focuses on the possible alteration of auxin level in organs of plants differing in sensitivity to a lack of oxygen. It reveals the importance of exogenous IAA treatment or its overproduction in transgenic plants as well as the means by which it regulates plant survival under conditions of oxygen deprivation.

## 2. Materials and Methods 

### 2.1. Plant Material and Growing Conditions

Seeds of spring wheat (*Triticum aestivum* L. cv. Leningradka) were purchased from Suida Breeding Station (Leningrad Region, Russia) and seeds of japonica rice (*Oryza sativa* L. cv. Liman) were obtained from the All-Russian Rice Research Institute (Krasnodar, Russia). The seeds were surface-sterilized with 5% NaClO solution (*v*/*v*) for 15 min, washed several times in distilled water, and soaked for 1 h in hot water at 45–50 °C for wheat and at 50–55 °C for rice. After germination for two days in the dark at 23 °C for wheat and at 28 °C for rice, the seedlings were planted in perforated plastic plates of beakers filled with continuously aerated Knop nutrient solution (0.2 strength) and grown at an irradiance of 100 W/m^2^, at a photoperiod of 14 h (for wheat) and 12 h (for rice) at 22–25 °C as described previously [41]. 

Seeds of wild type tobacco (*Nicotiana tabacum* L. cv. Wisconsin 38) and transgenic IAA-overproducing lines 16:15, 18:13 and 1 × 2a were kindly supplied by Prof. Folke Sitbon (Uppsala BioCenter, Swedish University of Agricultural Sciences, Uppsala, Sweden). In this study we used transgenic lines created by crossing primary transformants expressing the *Agrobacterium tumefaciens* IAA biosynthesis genes *iaaM* and *iaaH* under control of 35S promoters. 35S:*iaaM* × 35S:*iaaH* plants accumulate 2–3 times higher auxin in leaves and stems than the wild type [49,50]. Tobacco seeds were first surface-sterilized with 70% ethanol (*v*/*v*) for 5 min, washed several times with sterile water, subsequently sterilized with 10% NaClO solution (*v*/*v*) for 15 min, washed again several times with sterile water and germinated in Petri dishes on a solid MS basal medium [51] supplemented with sucrose (20 g/L) and Gelrite™ (Duchefa Biochemie, Haarlem, The Netherlands; 30 g/L). After a week seedlings were transferred to the same medium in 1 liter sterile glass jars (3 plants per jar) and grown for another 3-4 weeks at a temperature of 22–25 °C with a 16 h photoperiod. 

### 2.2. Imposition of Anoxic and Post-Anoxic Conditions, IAA Treatment

For measurement of endogenous auxin content a part of the cereal seedlings were analyzed as the initial material (time point 0), and the other seedlings were separated into control and experimental groups. Each set of twenty seedlings were placed in glasses containing 20 mL of Knop nutrient solution (0.2 strength). Glasses were placed into the chamber through which gaseous nitrogen containing less than 0.01% of oxygen was purged for 45 min in order to create anaerobic conditions. Then, the chambers were hermetically closed and put in the dark in order to prevent oxygen formation in the light. Anaerobic conditions were checked using Anaerotest^®^ indicator (Merck, Darmstadt, Germany). Duration of anoxic exposure was 12 h. Control seedlings were kept in dark at normal oxygen level (21%). For modeling post-anoxia anaerobic chambers were opened and plants were transferred into aerobic atmosphere for 1, 6, 24, 72 and 144 h after exposing to anoxia. After 24 h of reoxygenation plants were exposed to light.

For investigation of auxin effects on plant viability and oxidative processes seedlings were treated with 10 μM IAA dissolved in Knop nutrient solution (0.2 strength) for 1 day in the dark before the imposition of anoxia, i.e. the hormone was supplied via roots. In preliminary experiments, concentrations of IAA were tested in the range from 10^−8^ to 10^−4^ M, and 10^−5^ M (10 µM) was the most effective (data not shown).

When testing anoxia effect on IAA-overproducing tobacco sterile glass jars with plants were opened, placed into the anaerobic chamber and flushed with gaseous nitrogen as described above. The duration of anoxic treatment was 4 hours. Control plants were kept at the open air.

### 2.3. Quantification of Endogenous IAA

IAA was extracted, purified and quantified by ELISA as previously described [41] with some modifications. Shoots and roots of ten seedlings (0.3–1 g of fresh weight (FW)) were ground in liquid nitrogen. Thoroughly homogenized plant material was transferred into centrifuge tubes with 10 volumes of cold 80% methanol (*v*/*v*) containing 400 mg/mL of sodium diethyldithiocarbamate, and Polyclar AT (Serva, Heidelberg, Germany; 10% based on the plant the material weight). The extraction was performed overnight at 4 °C. After centrifugation at 12,000× *g* for 20 min the supernatant was collected and the pellet was washed three times for 30 min with 1/2 of the initial methanol volume. The combined extract was passed through the Sep-Pak C18 column (Waters, Milford, MA, USA) two times and IAA was eluted from the column with 10 mL of 80% methanol (*v*/*v*). Then methanol was evaporated to water phase in the refrigerated CentriVap centrifugal concentrator (Labconco, Kansas City, MO, USA) at 10 °C, and the water phase was diluted to 3 mL with deionized water, and pH was adjusted to 2.8–3.0 with 1 N HCl. Further IAA was extracted three times with equal volumes of ethyl acetate. The organic phase was collected and combined extract was evaporated to dryness in CentriVap (Labconco). Dried samples were dissolved in 100% methanol and IAA was subsequently purified by TLC. Separation was performed for 1 h in isopropanol:28% ammonia:water (10:1:1, *v*/*v*/*v*) system in Silufol-254 UV plates (Kavalier, Votice, Czech Republic). IAA bands (Rf 0.35–0.40) were scraped off from chromatography plates into micro tubes and extracted with 70% ethanol (*v*/*v*). IAA was methylated with diazomethane produced from nitrosomethylurea. Alcoholic solutions of Met-IAA were used for the solid-phase immunoenzyme analysis. Solution of Met-IAA produced by diazomethane methylation of a known amount of IAA was used as a standard. Immunoassay of auxins was performed with reagents obtained and characterized as described by Veselov et al. [52]. The reagents were kindly provided by Prof. Stanislav Yu. Veselov (Bashkir State University, Ufa, Russia) and Prof. Farida M. Shakirova (Institute of Biochemistry and Genetics, RAS, Ufa, Russia). ELISA was performed with standard flat bottom polystyrene 96-well plates (Sarstedt, Nümbrecht, Germany). Measurements were done by ELISA analyzer FFM-1 (Cortek, Moscow, Russia) at 492 nm. The IAA content in plant tissues was determined from the standard curve and calculated in pmol per g FW. 

### 2.4. Electrolyte Leakage Test

Electrolyte leakage was measured from shoots and roots of control, anoxic and IAA-pretreated anoxic plants. Collected shoots and roots from 10 seedlings were placed separately in the test tubes and incubated in 10 mL of deionized water for 5 h in dark at room temperature [53], then electrical conductance (EC) was measured using conductivity meter HI2300 (Hanna Instruments, Woonsocket, RI, USA). Thereafter, plant samples were boiled in the same water for 30 min to induce total leakage and cooled to room temperature before measurement. Relative electrolyte leakage was calculated according to the formula:

EL (%) = (EC_L_/EC_T_) × 100% (1)

where EC_L_ is the electrical conductance of leaked electrolytes at room temperature, and EC_T_ represents the total electrical conductance after boiling.

### 2.5. Evans Blue Staining

The level of cell damage in tobacco after anoxic treatment was measured by Evans blue staining assay [54,55]. Ten leaf discs (10 mm diameter) were weighed and incubated with 3 mL 0.05% Evans blue (*w*/*v*) in 20 mM MES (pH 5.8) on the shaker at 100 rpm for 30 min at room temperature. After the incubation the discs were washed three times with 20 mM MES to remove excess of Evans blue dye, placed into 2 mL micro tubes, ground with 1 mL of 1% SDS (*w*/*v*) in 50% methanol (*v*/*v*), and incubated for a further 30 min at 50 °C to release the absorbed dye from the plant tissues. Following centrifugation at 9000× *g* for 5 min, optical density of supernatant was measured at 600 nm (U-2000 spectrophotometer, Hitachi, Tokyo, Japan). The cell damage was calculated in µmol of Evans blue absorbed by plant tissues per g of FW.

### 2.6. Thiobarbituric Acid Reactive Substances Assay

Level of lipid peroxidation was evaluated using thiobarbituric acid as described earlier [56]. Roots of ten seedlings (about 1 g) were thoroughly homogenized with quartz sand, mixed with 5 mL of 0.1% TCA (*w*/*v*). After filtration 0.5% thiobarbituric acid (*w*/*v*) in 20% TCA (*w*/*v*) was added to an aliquot of the filtrate, then the mixture was incubated in a boiling water bath for 30 min. After cooling and centrifugation at 8000× *g* for 20 min, absorbance of the supernatant was measured at 532 nm (UNICO 2800H UV/VIS spectrophotometer, Dayton, NJ, USA). The amount of thiobarbituric acid reactive substances (TBARS) was calculated per g FW using malondialdehyde extinction coefficient (0.156 × 1/(μ*M* × cm)) [57].

### 2.7. Xylenol Orange Assay

Hydrogen peroxide production was measured using the xylenol orange assay [58] after the incubation of plant material (roots of 10 seedlings) in 15 mL of 50 mM phosphate buffer (pH 6.0) for 30 min. The working reagent contained 0.1 mL of 25 mM FeSO_4_ and 25 mM (NH_4_)_2_SO_4_ in 2.5 M H_2_SO_4_, and 10 mL of 125 µM xylenol orange and 100 mM sorbitol [59]. The assay mixture consisted of 0.1 mL of the incubation solution and 3 mL of the working reagent. After 30 min in the dark absorbance was measured at 560 nm (UNICO 2800H UV/VIS spectrophotometer). The amount of H_2_O_2_ produced was determined from a standard curve and calculated in nmol per g FW per min. The specificity of the assay was confirmed by the inhibition of hydrogen peroxide production by 500 units of catalase.

### 2.8. Statistics

Data on the figures are presented as mean ± SE for ≥ 5 experiments. Analysis of variance was done with GraphPad Prism 5 for Windows).

## 3. Results

### 3.1. Effects of Anoxia and Reoxygenation on IAA Content in Wheat and Rice

**Figure 1:**
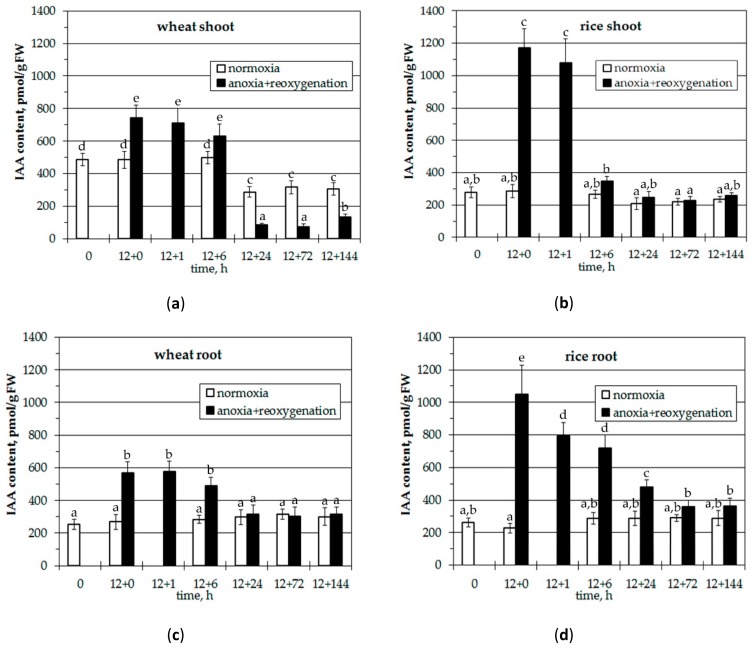
Effect of anoxia (12 h) and reoxygenation (+1 to +144 h) on IAA content in shoots (**a**, **b**) and roots (**c**, **d**) of wheat (**a**, **c**) and rice (**b**, **d**) seedlings. Values with the different letters are significantly different at *p* < 0.05, according to post-hoc LSD test.

Before the imposition of anoxia (time point 0), shoots of wheat seedlings contained about twice as much IAA as the roots and rice shoots (Figure 1). 

Rice roots and shoots accumulated about the same amount of free IAA. 12 h of anoxic treatment (12+0 point) led to a rise of IAA level by 50% in wheat shoots (Figure 1a) which was maintained until 6 h of reoxygenation followed by a significant decrease to about 20%–30% of normoxic and 10% of anoxic level. An anoxia-induced increase of IAA levels in wheat roots was twofold (Figure 1c). After 24 h of reaeration it returned to initial/control normoxic level and was maintained until the end of the experiment. Shoots and roots of rice seedlings reacted to anoxia and subsequent aeration in a similar manner (Figure 1b,d). Oxygen deprivation brought about significant accumulation of free IAA (4-fold in shoots and 5-fold in roots) followed by decrease of hormone content to normoxic levels at 6 h of reoxygenation in shoots and 72 h in roots.

Thus, 12 h of anoxia led to an increase of auxin content in plant tissues, especially in rice roots and shoots. Rice roots accumulated higher amounts of hormone during anoxia and restored its initial before-stress level in shoots at reaeration while in shoots of wheat seedlings it was reduced compared with normoxia. The level of IAA returned mainly to normoxic level both in roots of wheat and rice seedlings during reoxygenation.

### 3.2. Effects of Pretreatment with IAA and IAA Overproduction on Anoxia-Induced Plant Damage

**Figure 2:**
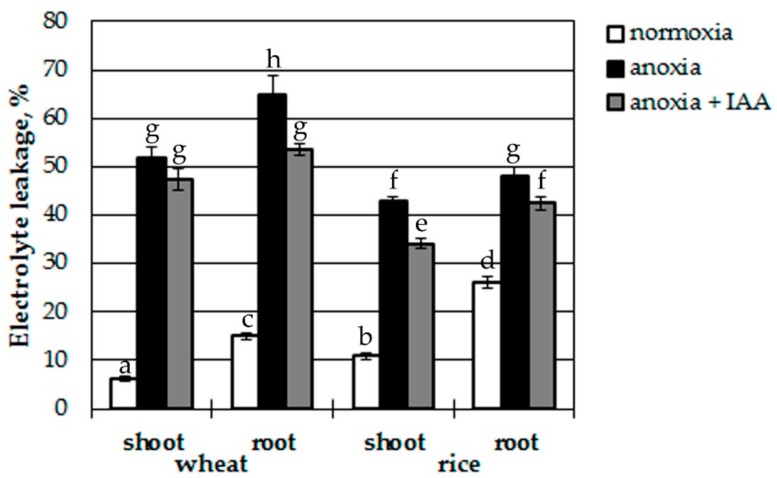
Effect of treatment with IAA (10 μM) on anoxia-induced electrolyte leakage from wheat and rice seedlings. Values with the different letters are significantly different at P < 0.05, according to post-hoc LSD test.

In order to determine whether IAA accumulation in plant tissues during short-term anoxia does or does not have a protective action on plant survival, wheat and rice seedlings were treated with 10 µM IAA administrated via root system before anoxic exposure. After 12 h of anoxia the relative electrolyte leakage (EL) was measured form shoots and roots of normoxic, anoxic and IAA-pretreated anoxic seedlings (Figure 2). Electrolyte leakage assay evaluates the integrity of cell membranes and a higher leakage value corresponds to higher degree of cell damage.

Surprisingly, the level of EL form tissues of wheat seedlings was lower than from rice seedlings at normoxic conditions. EL from roots was 2–2.5 times higher than from shoots of both plants. The effect of anoxia resulted in drastic stimulation of EL from wheat seedlings (8-fold in shoots and 4-fold in roots). Auxin pretreatment had no significant effect on anoxia-induced EL from wheat shoots, but reduced damage in wheat roots by 18%. In rice seedlings anoxia led to only 4-fold increase of EL in shoots and 2-fold in roots reflecting a higher tolerance of rice to oxygen deprivation. IAA treatment prior to anoxia lowered EL by 10%–15% in shoots and roots of rice seedlings. Thus, IAA treatment favors membrane integrity during anoxia.

**Figure 3:**
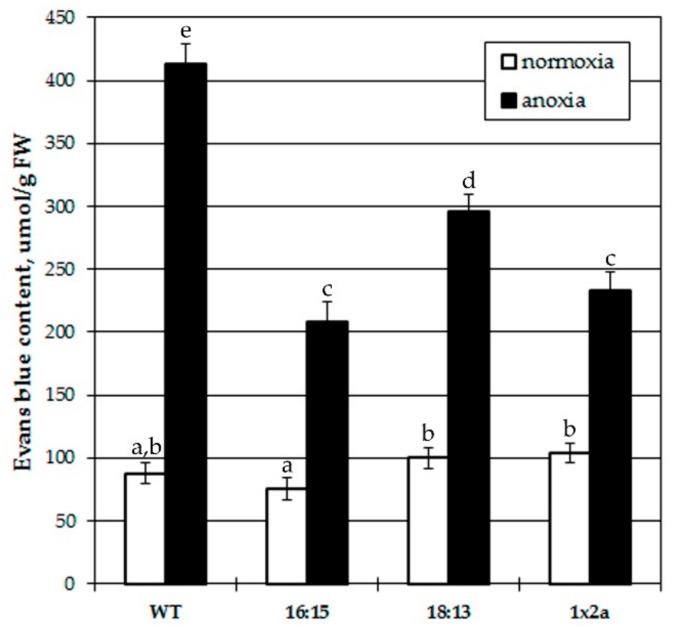
Effect of anoxia on cell damage in wild type (WT) tobacco and 35S:*iaaM* × 35S:*iaaH* lines (16:15, 18:13 and 1 × 2) overproducing IAA. Values with the different letters are significantly different at *p* < 0.05, according to post-hoc LSD test.

Data on treatment with exogenously supplied hormones are not always relevant since part of the hormone can be degraded or not absorbed and the administration through the root system affects its translocation within plant. Therefore, we used tobacco plants with endogenously elevated IAA level. 35S:*iaaM* × 35S:*iaaH* plants exhibited leaf curling, epinasty and intensified formation of adventitious roots at stem and petioles due to accumulation of 2–3 times higher IAA in leaves and stems than the wild type [49,50]. Evans blue staining assay was carried out to evaluate the possible effect of high auxin content on anoxia-induced cell damage (Figure 3). Evans blue dye enters only in dead cells with a freely permeable plasma membrane, therefore, a higher staining level corresponds to higher damage. Staining with Evans blue was similar in all tested tobacco plants, wild type and IAA-overproducing transgenes at normoxia. Imposition of 4 h anoxia brought about significant increase of dye absorption in all plants too, but it was about 5-fold in wild type plants, whereas in 35S:*iaaM* × 35S:*iaaH* lines it did not exceed 3 folds, i.e. all IAA-overproducing plants were less damaged by anoxia. 

### 3.3. Effects of Plant Pretreatment with IAA on Oxidative Processes after Anoxia

**Figure 4:**
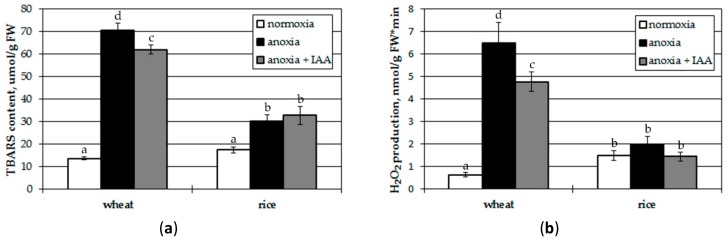
Effect of treatment with IAA (10 μM) on lipid peroxidation (**a**) and hydrogen peroxide production (**b**) in the roots of wheat and rice seedlings after 12 h anoxia. Values with the different letters are significantly different at *p* < 0.05, according to post-hoc LSD test.

The viability tests used in this study, such as electrolyte leakage and Evans blue staining, reflect cell membrane integrity, which may be disturbed by lipid peroxidation due to production of reactive oxygen species, e.g., H_2_O_2_. Therefore, the effects of plant pretreatment with IAA on lipid peroxidation and H_2_O_2_ production at oxygen deprivation were assessed as well (Figure 4). We tested roots of seedlings for several reasons: (1) during treatment the hormone enters roots first, (2) since IAA treatment showed significant effects on the roots of both tested plants (Figure 2), and (3) since H_2_O_2_ production is easy to measure in roots.

Anoxia led to a 5-fold increase of TBARS content in roots of wheat seedlings, corresponding to the lipid peroxidation level, and application of 10 µM IAA suppressed it by 13% (Figure 4a). In roots of rice seedlings a lack of oxygen provoked only 2-fold stimulation of lipid peroxidation which was unaffected by IAA treatment. 

Anoxic treatment in wheat led to a 10-fold increase of hydrogen peroxide production (Figure 4b), and IAA reduced it by one third. There were no significant changes found in H_2_O_2_ production in rice roots during anoxia and post-anoxic reoxygenation. Thus, IAA pretreatment prior to anoxia provided inhibition of destructive oxidative processes in wheat seedlings, but had no effects on rice.

## 4. Discussion

The obtained data showed a significant accumulation of free IAA in plant tissues during 12 h of anoxia (Figure 1). There was 1.5–2-fold rise of IAA level in wheat seedlings and 4–5-fold increase in rice. Earlier we reported a similar IAA accumulation in cereal seedlings at short-term (6–12 h) anoxia [41]. After the first transient peak of accumulation, IAA level decreased to initial normoxic level in wheat seedlings and continued to increase in rice finishing to 5–7-fold level after 3 days of anoxia [41]. A similar auxin content dynamics in rice after 1–3 days of anoxia was reported by Mapelli et al. [40]. A medium hypoxia tolerant oat demonstrated an intermediate IAA level between sensitive wheat and tolerant rice in oxygen-deprived environment [38,39]. The present data testify that plants resistant to anoxia are characterized by a higher level of anoxia-induced IAA. The mechanisms of IAA accumulation are still enigmatic. Different pathways of biosynthesis, polar transport, conjugation and degradation are supposed to regulate IAA concentration. It is unlikely that oxygen deprivation intensifies auxin synthesis, since synthesis directly depends on molecular oxygen. It might block the reversible β-oxidation of indolebutyric acid responsible for the generation of active IAA [60]. Another way of IAA over accumulation during oxygen deficiency may be suppression of oxidative breakdown. We reported inactivation of IAA oxidase being more severe for tissues of medium tolerant oat seedlings [38]. Another enzyme of auxin degradation is DIOXYGENASE FOR AUXIN OXIDATION (DAO) converting IAA to oxIAA. Alteration of the activity of DAO owing to mutations leads to a phenotype with elevated auxin levels. Both reactions are provided by oxidation and supposed to be down-regulated during anoxia. However, the intensity of this down-regulation might depend on tissue origin and sensitivity of plant species to the lack of oxygen [61]. Additional mutations in DAO resulted in upregulation of gene encoding GH3s family responsible for IAA conjugation, which makes the system even more complex. The auxin level in plant tissues may also be changed via redistribution of PIN transporters. Stimulation of expression of genes encoding PIN1-2, PIN2 and IAA-GH3 was reported during induction of adventitious roots in *Solanum dulcamara* under partial and complete submergence [62]. It is worth noting that the level of auxin did not change.

During further reoxygenation the IAA level decreases rapidly in shoots and gradually in roots of both resistant and sensitive plants (Figure 1). Perhaps this is due to the higher availability of oxygen in shoots, where auxin is rapidly metabolized through the activation of different peroxidases by ROS under conditions of post-anoxia [63]. Note that in sensitive wheat seedlings the decrease in IAA content was more prolonged in shoots during reoxygenation, while in rice it rapidly recovered to normoxic level. Probably this effect might be an indicator of plant facility to tolerate oxygen shortage. 

The EL test showed higher tolerance of rice seedlings than wheat ones, since they were less damaged (Figure 2). Treatment of seedlings with exogenous IAA reduced cell damage measured by EL tests in roots of both plants tested and in shoots of rice seedlings (Figure 2). In wheat shoots, the auxin effect was insignificant, and it was distinguished by a lower level of IAA during reoxygenation, again pointing out a lesser degree of oxygen-deficiency tolerance. The IAA treatment also provided inhibition of destructive oxidative processes (lipid peroxidation and hydrogen peroxide production; Figure 4) in wheat seedlings while it had little effect on rice. In rice the anoxia-induced oxidative processes were undistinguishable from normoxic control both in auxin-treated and auxin-untreated seedlings. This could be a reflection of higher tolerance on the part of rice to oxygen deprivation, or it could be the result of a higher endogenous IAA content.

Over the last decade there has been a wide spectrum of investigations concerning IAA and ROS interaction. Usually, IAA treatment stimulates ROS production and ROS production, in turn, intensifies oxidative IAA breakdown [64,65,66,67,68]. Our data on IAA treatments contradict these findings; but there are also reports showing the opposite effect of IAA treatment. IAA application to maize roots resulted in inhibition of growth that was accompanied by a reduction of ROS production [69]. Hormone-induced elevation of tolerance to different abiotic stresses was demonstrated for cytokinines [70]. Recently rice seedlings were shown to increase adaptation after exogenous salicylic acid and H_2_O_2_ application at osmotic stress [71].

Results from plant treatment with exogenous IAA inspired us to test tobacco plants with endogenously elevated IAA level. 35S:*iaaM* × 35S:*iaaH* plants contained 2-3 times higher IAA in leaves and stems than the wild type [49,50]. All tested IAA-overproducing tobacco plants were less damaged by anoxia than the wild type plant (Figure 3), endorsing a positive IAA role in providing plant survival during oxygen shortage and subsequent reoxygenation.

Another effect of auxin is to promote the growth of coleoptiles, both by itself and by triggering the synthesis of ethylene [72]. In some species, auxin, more than gibberellins, is required for ethylene-mediated growth response to submergence conditions [32]. Nevertheless, a number of studies have shown that exogenous IAA does not affect the elongation of isolated coleoptiles and intact rice seedlings [36,73]. IAA participates in metabolic regulation during oxygen deficiency. Treatment with IAA increases ADH activity in lettuce [74], protein synthesis and accumulation of solutes in rice coleoptiles [75]. Auxin affects hyponastic response [33] and stimulates adventitious rooting [23].

Such a variety of feedback is commonly associated with alteration in plant cell sensitivity to the hormone. The auxin signaling pathway starts with receptors such as TRANSPORT INHIBITOR RESPONSE1 (TIR1) [76]. Further ubiquitination of Aux/IAA proteins results in release of ARF transcription factors. The involvement of auxin transduction elements in regulation of adaptive reaction on oxygen deprivation is in agreement with recently discovered data.

Hypoxia was found to inhibit lateral root formation due to both the IAA12-ARF5 auxin module and the IAA14-ARF7-ARF19 module [18]. It is known that the *SAUR39* gene encoding small auxin-up RNA was shown to be sensitive to anoxia. It acts as a negative regulator of auxin biosynthesis and transport in rice coleoptiles. Overexpression of the *SAUR39* gene leads to a reduction of free IAA levels and lower auxin transport in rice, which results in shoot shortage [77]. The signaling networks are realized not only at the transcriptional, but also at the microRNA level. miR160, miR164, miR167, miR171, miR390, and miR393 were found to be conserved within tested plant species and involved in auxin homeostasis and signal transduction [78]. Recently, miR164, miR167, and miR390 were found to decrease expression, whereas miR160 leads to its elevation in wild tomato roots treated with hypoxia [79].

Thus, the increase in the endogenous level of IAA can be considered specific to plant species and organ/tissues tolerant to oxygen deprivation. Other responses might be connected to alterations in sensitivity to the hormone and indicate that the auxin effect is a step in a complicated adaptation to flooding. This causes a minor damage effect under lack of oxygen and raises the plant tolerance. However, the mechanism of this hormone effect is still under investigation.

To date, there is no data on the role of IAA in post-anoxic conditions. Our experiments revealed a rapid decrease of shoot IAA level after plants were placed back to normoxia. The caused decrement in growth is apparently also a part of the adaptive mechanism, which allows the plant to exit the emergency growth mode when it reaches atmospheric air, and the plant switches to overcome the effects of oxidative stress, restores normal metabolic processes and continues to live under normal conditions without significant losses. The gradual restoration of the IAA level in the roots allows the plant to restore the level of root growth, not immediately upon returning to normal aeration conditions, but after a period of reparation after stress exposure.

Similar tendencies are observed in wheat plants, but they are less pronounced and, apparently, do not provide sufficient tolerance, since even after 6 days under normal conditions, wheat does not restore the initial level of the hormone content in shoots. Thus, a tolerant plant under conditions of both anoxia and early post-anoxic aeration differs from sensitive plant with an increased level of auxin. 

## 5. Conclusions

In the tolerant rice plant, IAA accumulated during anoxia. This phenomenon was repeatedly shown in earlier studies and confirmed by this study. During the post-anoxic period there was a quick return to the initial level of hormone content. This may indicate the importance of hormonal system in overcoming both anoxia and post-anoxia. In the sensitive wheat plant, the hormone accumulation at anoxia was not so pronounced, and reoxygention led to a decrease of hormone level compared with the control. This probably indicates an ineffective function of the hormonal system or hormone utilization in sensitive plants. A treatment with exogenous auxin was found to be most effective for wheat in increasing its resistance to anoxia and oxidative post-anoxic stress. Exogenous IAA treatment enhanced plant survival under anoxia by decreased electrolyte leakage, production of hydrogen peroxide and lipid peroxidation. The positive effect of external IAA application was confirmed with improvement of tolerance to oxygen deprivation in the 35S:*iaaM* × 35S:*iaaH* IAA-overproducing tobacco lines.

Auxin is an important regulator of a wide spectrum of processes. The obtained data emphasize the specific role of auxin in plant tolerance to anoxia and further reoxygenation. The mechanisms behind its function are still puzzling, but the involvement of regulation at several levels (transcription, level of RNA, alteration in activity of many enzymes and finally growth and metabolism, among others) allows us to suggest, that this role is a special one.
